# Evaluation of physicochemical properties and enzymatic activity of organic substrates during four crop cycles in soilless containers

**DOI:** 10.1002/fsn3.757

**Published:** 2018-10-19

**Authors:** Pedro A. Mejia Guerra, Maria del Carmen Salas Sanjúan, Maria J. López

**Affiliations:** ^1^ Doctoral Program in Protected Agriculture Almeria University Almeria Spain; ^2^ Department of Agronomy Almeria University Campus de Excelencia Internacional Agroalimentario, ceiA3 La Cañada Almeria Spain; ^3^ Unit of Microbiology Department of Biology and Geology CITE II‐B Universidad de Almería Campus de Excelencia Internacional Agroalimentario, ceiA3 Almeria Spain

**Keywords:** coconut fiber, dehydrogenase, glucosidase, organic matter, substrate reuse, vermicompost

## Abstract

**Background:**

Organic soilless production in containers requires substrates with appropriate physicochemical and biological properties to ensure that production is sustainable and profitable for several production cycles. The main objective of this study was to comprehensively evaluate these properties in three different mixtures of organic substrates (vermicompost [V] and coconut fibers [CF] in ratios 20V80CF, 40V60CF, 60V40CF) for four horticultural crop production cycles (PCs) using vermicompost tea (VT) as the main source of nutrients.

**Results:**

Readily available water (25%) in the control treatment (20V80CF) was below the recommended limit, and dry bulk density (>450 g/L) surpassed the recommended limit in the 60V40CF treatment (*p* < 0.05). In terms of chemical properties, cations and anions in the saturated media extract decreased significantly to values below established optimal conditions. Furthermore, the substrates presented high enzymatic activity in successive production cycles (*p* < 0.05), including dehydrogenase (350–400 μg TFF g^−1^), acid phosphatase (4,700 μg p‐nitrophenol g^−1^ soil hr^−1^), and β‐glucosidase (1,200 μg p‐nitrophenol g^−1^ soil hr^−1^) activity during transformation from organic matter to inorganic compounds.

**Conclusion:**

The 40V60CF treatment presents adequate physicochemical and biological characteristics for reuse for more than four growing cycles when organic supplements are administered.

## 1 INTRODUCTION

The growing global demand for food to supply the increasing world population, without compromising natural resources for future generations, represents one of the greatest challenges to agricultural science. The current model of intensive agriculture is causing severe effects on global climate change, and therefore, urgent transformation is required in agricultural and livestock production systems to reduce negative impacts on the environment and human health (FAO [Food and Agriculture Organization of the United Nations], 2017). The use of renewable or sustainable organic substrates (vermicompost, compost, coconut fiber, wood fiber) in containers has become an economically and environmentally sustainable production alternative in recent years as a result of the beneficial physicochemical and biological properties and their lower environmental impact and greater sustainability than inert (perlite, rockwool, polystyrene foam) and noninert (volcanic stone, vermiculite) inorganic substrates (Burnett, Mattson, & Williams, 2016). Despite being the most widely used organic substrate in the world, sphagnum peat is facing a serious problem in that it is not renewable. Thus, sphagnum peat is an unsustainable substrate in the consideration of several government strategies (nonproducer countries), and peat should be replaced by more sustainable organic substrates (Schmilewski, 2017). One strategy involves the use of plant residues, which can be used as biofertilizers. This technique has been used in ecological agricultural production systems to cultivate crops without soil (Lim, Wu, Lim, & Shak, 2015). It has been demonstrated that organic substrates such as vermicompost (V) and coconut fiber (CF) can be successfully used in soilless containers (Vo & Wang, 2014). In addition, vermicompost displays a number of nutritional advantages that are related to its formation process. It is generated by a process called vermicomposting, which consists of bio‐oxidation and transformation of organic matter (OM) through the combined action of worms (*Eisenia fetida*) and microorganisms under aerobic and mesophilic conditions (Kiyasudeen, Ibrahin, Quaik, & Ismail, 2016). Furthermore, N‐fixing and P‐solubilizing bacteria, which improve the nutritional characteristics of vermicompost, have been isolated from the gut of *E. fetida* (Hussain et al., 2016). In this context, new soilless production techniques must be improved and implemented with consideration of the particular characteristics of a substrate in a container with limited volumes of water, air, and nutrients available to the plant (Bowman & Paul, 1983). However, organic substrates (in comparison to soil) present several advantages for use as growth media and are potentially important for biofertilization, biocontrol, bioamendment, and bioremediation. Furthermore, organic substrates may have beneficial physicochemical and biological properties (Owen, Williams, Griffith, & Withers, 2015). In particular, physical characteristics are considered the most critical because they are difficult to modify once a crop is planted and because the water–air–roots relation should be maintained to ensure adequate plant growth and to prevent biotic and abiotic stressors (high salinity, hydric stress). Specifically, an adequate substrate should have the following characteristics: total porosity (P) of 70%–85%, air volume (AV) of 10%–20%, and readily available water (RAW) ≥30%. In organic substrates of either single or mixed materials, the physical characteristics are mainly determined by the high capacity of the substrate to retain water at low tensions (10–20 cm of water column) (FAO, 2018). Additionally, the substrate should have good drainage and adequate aeration capacity (Cabrera, 1999). The chemical characteristics also determine substrate quality, especially characteristics related to the nutrient balance and the efficient use of available nutrients. However, these characteristics can be easily modified, such as through the addition of additives to the nutrient solutions. Specifically, the most influential chemical characteristics are pH, temperature, electrical conductivity (EC), and cation and anion balance in the saturated media extract (SME) (Warnecke, 1986). The control of these factors is fundamental for ensuring adequate crop production. These factors can be monitored via different analysis techniques, including examination of the drainage in the substrate and plant sap (Pardossi, Incrocci, Salas, & Gianquinto, 2017). Several methods can be used to determine the chemical properties of substrates in containers. Two, in particular, have become popular: the SME and leachate methods. The SME method is widely used in university and commercial laboratories (Cabrera, 1999). The general guidelines for the interpretation of analyses are based on numerous studies and soil fertility tests (Warnecke, 1986). In addition, the biological characteristics of organic substrates can be determined via an analysis of the total microbiota or functional groups, such as solubilizers of phosphate, nitrogen fixers, and nitrifying and ammonifying agents. The measurement of soil (substrates) enzyme activities has the potential to provide unique integrative biological and biochemical assessments of soil (substrates) because of their relationship with soil biology and their rapid responses to all changes (i.e., anthropogenic, agronomic, chemicals, and weather conditions). This is the reason why there is a clear relationship between nitrogen content and urease, dehydrogenase (DHA), phosphatase, and β‐glucosidase (β‐GLU) (Qian et al., 2014). The quantity of nitrogen in organic substrates is influenced by two different microorganisms, N‐fixing bacteria and nitrifiers. The first microorganism converts atmospheric nitrogen (N_2_) into ammonium (NH_4_^+^), and the second oxidizes the ammonium (NH_4_^+^) to nitrites (NO_2_) and nitrates (NO_3_^−^). The biological–microbial activity of organic substrates can also be measured via analyses of enzymatic activity, including the determination of DHA, acid phosphatase (ACP), and β‐GLU activity (Cordovil et al., 2017). DHA is an oxidoreductase enzyme that is present only in viable microbial cells. DHA carries out the biological oxidation processes of organic compounds through dehydrogenation. ACP activity in the soils may originate either from plant roots or from microorganisms such as fungi and bacteria (Dinkelaker & Marschner, 1992). ACP hydrolyzes the organic phosphorous present in OM. β‐GLU is involved in the decomposition of cellulose compounds and is synthesized by soil microorganisms in the presence of suitable substrates (de Almeida, Naves, & da Mota, 2015).

Considering the above, within the contextual framework of sustainable agriculture, the main objective of this research was to evaluate the physicochemical and biological properties of three mixtures of organic substrates during the production of four consecutive crops and to estimate the reuse potential of these mixtures.

## 2 EXPERIMENTAL

### 2.1 Experimental design and treatments

The experiment had a completely randomized block design with four replications comprising three containers each (12 plants total); the treatments were established according to the percentage of the mixture by volume of each organic material used as the substrate (V, CF). Two factors were considered: substrate (V, CF) (% v:v) and production cycle (PC). There were three substrate treatments: 40V60CF (% v:v), 60V40CF (% v:v), and 20V80CF (% v:v) (control); the latter treatment was considered the substrate control treatment, as many references have concluded that a rate of 20V80CF can be included without adversely affecting plant performance (Maher & Prasad, 2001; Pronk, 1995). The mixtures were based on volume (% v:v). The initial characteristics of the CF and V used in the substrate mixture treatments are presented in Table 1.

**Table 1:** Characteristics of substrates: coconut fiber (CF), vermicompost (V)

Initial parameters	CF	V
Particle size (mm)	0–12	<5
Solid particle density (g cubic centimeter^−1^)	0.1	0.77
Total porosity (%)	95.4	67.9
pH	5.8–6.8	7.73
Cation exchange capacity (mmol 100 g^−1^)	60–130	25–30
Electrical conductivity (EC), dS/m	<0.7	0.9
Organic matter (% s/DM)	94.7	<15
Organic carbon (g/kg)	78.6	82.4
Organic nitrogen (g/kg)	1.8	9.9
Ratio C:N	43.66	8.32
Humic and fulvic acids (% w.w.)	ND	17.50

*Note*

ND: not detected.

The second factor was the PC. This factor had five treatments: melon (*Cucumis melo* L*. var. reticulatus*) (PC‐1), tomatoes (*Solanum lycopersicum* L.) (PC‐2), melon (*C. melo var. reticulatus*) (PC‐3), and lettuce (*Lactuca sativa* L.) (PC‐4). PC‐0 refers to the starting time (before transplanting).

### 2.2 Description of production cycles

Crops were selected according their resistance to cold weather in the zone of production (Almeria, Spain). Tomato crop was planted during autumn‐winter; melon crop, during spring‐summer; and lettuce crop, in autumn. The main agronomic characteristics of each PC are presented in Table 2. The cropping system included tomatoes as staked plants and melon as creeping plants. After tomato harvest, all aerial parts of the plants were removed, and the roots were left inside the substrate. The new crop (melon) was planted over the same substrate, and finally, the lettuce crop was transplanted after the melon harvest.

**Table 2:** Agronomic description of production cycles (PC‐1, PC‐2, PC‐3, PC‐4)

Production cycle	Commercial variety	Area (m^2^)	Density (plant/m^2^)	Transplant	Harvest
*Cucumis melo* (PC‐1)	Brisa (HM Clause)	288	1.00	12 March 2016	15 June 2016
*Solanum lycopersicum* (PC‐2)	Ramyle RZ F1 (Rijk Zwaan)	288	1.25	17 September 2016	8 March 2017
*C. melo* (PC‐3)	Brisa (HM Clause)	288	1.00	15 March 2017	18 June 2017
*Lactuca sativa* (PC‐4)	Yacht RZ (79–504) Salanova^®^	100	12.00	16 October 2017	28 November 2017

### 2.3 Location and characteristics of the crop

The experiment was conducted in a 900‐m^2^ multitunnel‐type greenhouse with active climate control at the Experimental Farm of Almeria University (UAL‐ANECOOP) Foundation (36.861905‐2.282529), Retamar, Almeria (Spain).

### 2.4 Irrigation management and nutrition

The drip irrigation system used drippers with a flow of 4 L/hr, and automated irrigation was performed by determining the percentage of humidity available in the substrate using dielectric sensors (Decagon moisture sensors) installed in each irrigation block. These sensors also measured the EC and temperature. The parameter used to determine the beginning of each irrigation was the humidity (water) content of the substrate (SWC). The SWC threshold was 20%–30% according to the phenological stage of the crop.

The nutritional program was balanced for each crop with the application of vermicompost tea (VT), which was derived from horticultural vegetable residues and served as the main nutrient source (Pant, Radovich, Hue, Talcott, & Krenek, 2009). Table 3 shows the analysis of the tested inputs of irrigation water (IW) and VT.

**Table 3:** Chemical parameters of irrigation water (IW) and vermicompost tea (VT) used during production cycles

Parameters	IW	VT	Procedure
pH	8.38	7.85	Electrometry
EC (dS/m)	0.414	6.300	Electrometry
Sodium absorption relation (SAR)	4.8	3.2	Calculation
Chloride (mg/L)	153	716	Chromatography
Nitrate (mg/L)	5	670	Chromatography
Phosphates (mg/L)	<2.50	<13.0	Chromatography
Sulfates (mg/L)	9.9	1,435	Chromatography
Bicarbonates (mg/L)	63	123	Volumetric
Sodium (mg/L)	87	297	AAS
Potassium (mg/L)	4.7	671	AAS
Calcium (mg/L)	13.5	449	AAS
Magnesium (mg/L)	7.2	132	AAS
Iron (μg/L)	<10.0	907	ICP‐MS
Manganese (μg/L)	<1.0	47	ICP‐MS
Copper (μg/L)	<10.0	211	ICP‐MS
Zinc (μg/L)	<25.0	<25.0	ICP‐MS
Boron (mg/L)	0.6	1.7	Spectrophotometry

*Note*

AAS: atomic absorption spectrometry; ICP‐MS: mass spectrometer with inductively coupled plasma.

### 2.5 Parameters evaluated

Yield was the production obtained (kg/m^2^) in each PC. Yield was measured as fruit fresh weight for PC‐1, PC‐2, and PC‐3; in lettuce (PC‐4), yield was measured as leaf fresh weight.

### 2.6 Physicochemical and biological characteristics of the substrate

The physical and chemical characteristics were determined for each PC in all substrate treatments at the beginning (PC‐0) and at the end of each PC. All the samples were processed in a specialized and certified center for this analysis (Laboratorio Agroambiental FRAISORO, UNE EN ISO 17025, Spain). The analysis followed the methods of the UNE‐EN 13039: 2012 (OM): OM is the carbon fraction of a sample (5 g) free from water and inorganic substances and is taken as equal to loss on dry incineration at (450 ± 25) °C; the principle is that the test portion of the substrate (5 g) is dried at (103 ± 2) °C and then ashed at (450 ± 25) °C. The ash is determined as the residue on ignition. The OM is taken to be the loss of mass on ignition, and both are expressed as a percentage by mass of the dried sample (see complete protocol in supplementary documents).

UNE‐EN 13041: 2012 (bulk density [BD], AV, P and RAW): Regarding terms and definitions, AV is the part of the volume of a sample filled by air measured under the conditions specified (e.g., −10 cm = −1 kPa suction); dry BD is the ratio of the dry mass and volume of the sample in grams per liter; water volume is the part of the volume of a sample filled by water measured under the conditions specified (e.g., −10 cm water = −1 kPa suction). The principle is that the sample is saturated in water and equilibrated on a sand box at −50 cm water (−5 kPa) pressure head. The sample is then transferred into double‐ring sample cylinders, rewetted and equilibrated at −10‐cm water (−1 kPa) pressure head. After equilibration, the physical properties are calculated (an equation for each property) from the wet and dry weights of the sample in the lower ring; it is also optional to apply the −50 and −100‐cm water pressure heads, respectively (see the complete protocol in supplementary documents).

The C:N ratio was calculated by gas chromatography and a thermal conductivity detector (PerkinElmer^®^ EA2400). The concentration of cations and anions, EC, sodium adsorption ratio (SAR), and pH were determined in the SME prepared according to ionic chromatography (nitrates [NO_3_^−^], chloride [Cl^−^], sulfates [SO_4_^2−^], phosphates [PO_4_^3−^]), atomic absorption spectrometry (calcium [Ca^2+^], magnesium [Mg^2+^], potassium [K^+^], sodium [Na^+^]), electrometry (pH, EC), calculation (SAR), and a specific sensor electrode to ammonium (NH_4_^+^). The substrate samples were taken at a depth of 10 cm inside the container, and a paste was made using substrate and water (as an extracting solution) at a dilution ratio of 1:2. Then, the liquid portion was separated from the solid portion for pH, EC, and main cation and anion analyses. All samples were processed in a lab certified to perform this type of test (Laboratorio Analitico Bioclinico LAB, UNE EN ISO/IEC 17025, Spain).

The biological activity in the substrates was measured by analysis of DHA, ACP, and β‐GLU enzymes. Three replicates were taken for each enzymatic activity, and DHA was determined according to the protocol established by Casida (1977). ACP was measured according to the protocol established by Tabatabai and Bremner (1969). β‐GLU (hydrolase or cellobiase) was determined according to the protocol of Tabatabai and Bremner (1969).

### 2.7 Data analysis

The substrate treatments (3) were crossed with the production cycle treatments (5), and statistical analysis was performed using Statgraphics Centurion XVII‐X64 (StatPoint, Inc., VA) software, and multivariate analysis of variance (ANOVA) was carried out using Fisher's comparison test of means, with the statistically least significant difference being expressed as *p* < 0.05 (LSD). Principal component analysis (PCA) was carried out to determine the variability, correlations, and synergisms among the different variables.

## 3 RESULTS AND DISCUSSIONS

### 3.1 Yield

The yield of each PC from PC‐1 to PC‐4 is shown in Table 4. Significant differences at the substrate level were found in PC‐1, PC‐2, and PC‐3 but not in PC‐4. Higher yields were obtained in the substrates containing higher proportions of vermicompost (40V60CF and 60V40CF) (*p* < 0.05). These results coincide with those of some authors stating that in *C. melo*, a higher proportion of vermicompost presented more cations and anions; as a result, plants have enough available nutrients for growth and production (Vo & Wang, 2014). Joshi, Singh, and Adarsh (2015) also stated that the nutritional richness present in vermicompost is fully available for plants as long as they have an adequate balance or percentage in their ability to exchange cations. This issue was also reported by Wang, Zhao, Zhang, Zhang, and Yang (2017), who evaluated the productivity, quality and levels of NO_3_^−^ and NH_4_^+^ between conventional and compost treatments and concluded that vermicompost presented the highest concentrations of NO_3_^−^ and NH_4_^+^. Therefore, vermicompost can be recommended as a fertilizer in crops. The yield of each PC was within an acceptable range (Guan et al., 2013), confirming that plants used nutrients from the VT and products of microbial action in the organic substrates with higher proportions of vermicompost (40V60CF and 60V40CF). Therefore, the highest yields were obtained for the 40V60CF and 60V40CF treatments (*p* < 0.05).

**Table 4:** Production per crop and substrate treatments

Substrates	PC 1 (kg/m^2^)	PC‐2 (kg/m^2^)	PC‐3 (kg/m^2^)	PC‐4 (kg/m^2^)
Control	4.60b	5.24b	3.71b	3.41a
40V60CF	5.17a	5.75a	5.10a	3.34a
60V40CF	5.20a	5.52ab	5.05a	3.42a

*Note*

Different letters indicate significant differences (Fisher's LSD) (*p < 0.05) in production per substrate treatment for each PC.

### 3.2 Physical properties of substrates

#### 3.2.1 Organic matter

**Figure 1:**
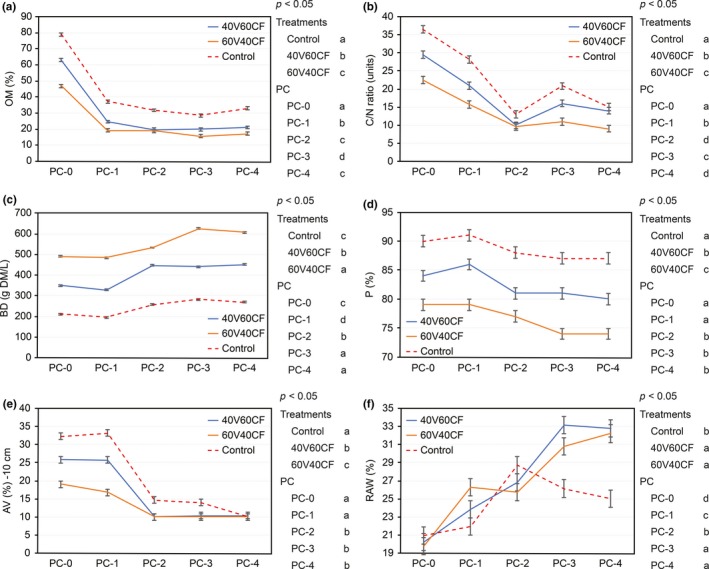
Physical properties between factor substrate treatments and production cycles (PC): (a) organic matter (OM); (b) ratio C:N; (c) dry bulk density (BD); (d) porosity (P); (e) air volume (AV); (f) readily available water (RAW). Different letters in the substrate legends indicate *p* < 0.05 between substrate treatments. Different letters in the PC legends indicate *p* < 0.05 between production cycles (*n* = 3). Error bars, Fisher's LSD test (95%)

Figure 1a shows significant differences (*p* < 0.05) in the OM content per substrate and PC. With respect to substrate, the control treatment had greater OM content across the four growing cycles, which is directly related to the higher percentage of CF in this substrate (20V80CF). As observed in Table 1, CF contains six times more OM than V. For PC, significant differences (*p* < 0.05) were found between PC‐0 and the subsequent cycles (PC‐1 to PC‐4). Once crops were cultivated in substrate (PC‐1), a strong reduction in OM followed, characterized by a decline from 62.82% (PC‐0) before transplantation to 24.90% at the end of the first cycle (PC‐1). The OM content remains stable in the remaining cycles (PC‐2, PC‐3, and PC‐4) at 21%–24%, which is below the reference value (>80%) suggested by Abad, Noguera, and Carrión (2004).

The above is explained based on the concepts defined by FAO (2017), which states that the main components of OM are micro‐organic fragments (>250 μm), microbial biomass, and humus, whose amounts reflect a dynamic equilibrium. In this research, substrates in PC‐0 contain fragments of macro‐OM that may be further transformed by microorganisms and taken up by the plant during the following cycles (PC‐1 to PC‐4); thus, OM is stabilized.

#### 3.2.2 C:N ratio

The C:N ratio showed significant differences (*p* < 0.05) per substrate and PC (Figure 1b). With respect to the substrate, the control had the highest ratio (C:N) at the beginning of production because of its higher percentage of CF (see Table 1). The 40V60CF substrate at PC‐4 and the control treatment presented C:N ratios greater than 10, which is considered acceptable and falls within the established optimal range (Casida, 1977). The C:N ratio in the PCs reached the lowest value at the end of PC‐2 (long cycle tomato) and tended to increase during PC‐3 and PC‐4, indicating that mineralization occurred. The final values (10–16) correspond to that of mature compost and are adequate for continued use. In general, a C:N ratio between 10 and 12 results in adequate nitrogen release, whereas ratios above or below this range may cause low or excessive nitrogen release, respectively. Carbon is important because its energy content takes the form of species such as carbohydrates, whereas nitrogen is essential for growth. Average C:N ratios vary among different organic substrates and their state of maturity. Thus, when OM is added to soil, the breakdown of the content by bacteria and fungi causes changes in the C:N ratio. It is important that any fertilizer added has sufficient nitrogen levels, or the addition will have a negative effect. The addition of composted manure, which typically has a C:N ratio of approximately 20:1, is desirable; however, the addition of sawdust, which has a high C:N ratio of 400:1, could be deleterious (Burnett et al., 2016). The microorganisms that break down the OM will very quickly run out of nitrogen and, therefore, will start to consume the nitrogen in the soil, which reduces the amount available to the plants and could depress crop yield (Cornell Composting Science & Engineering, 2018; PerkinElmer, Inc, 2018).

#### 3.2.3 Dry bulk density

The BD values showed significant differences (*p* < 0.05) with respect to the substrate and PC (Figure 1c). The general trend of BD was inverse of that reported for OM content. With respect to substrate, the control had the lowest BD, which is directly related to its higher CF content. Accordingly, 60V40CF yielded the highest BD because of its lower OM content and, as a result, greater ash content. According to the optimal level of BD suggested by Cabrera (1999), 60V40CF surpassed the optimal recommended range for BD (<450 g/L). Therefore, a high rate of compaction could be reflected in the poor development of root systems and lower yields (Nawaz, Bourrié, & Trolard, 2013). Regarding the BD values in the different PCs, an increasing BD tendency was observed from PC‐1 to PC‐2 (*p* < 0.05), which subsequently stabilized in PC‐3 and PC‐4. The BD values remained within an adequate range (<450 g/L) in 40V60CF and the control. These results demonstrate that BD is influenced by the OM content because increasing the OM content produces an increase in the total pore space, improving BD and porosity. This issue was reported by Bunt (1988), who stated that BD and total pore space are inversely related. For this reason, BD is also a determining factor of P, AV, and RAW.

#### 3.2.4 Porosity

Porosity also showed significant differences (*p* < 0.05) for both factors evaluated (Figure 1d). With respect to substrate, the control had the highest percentage of P, whereas the lowest percentage of P corresponded to 60V40CF. These results are directly related to the quantity of CF in the substrate mixture, which is inversely related to BD, as an increasing quantity of CF is associated with greater OM content. These findings coincide with those reported by Qi et al. (2018), who found that organic substrates favor the generation of organic compounds by roots, which help to form soil aggregates, reduce BD, and increase P. In the case of PC, an increase in P was found between PC‐3 and PC‐4 in 40V60CF and 60V40CF. In the control, P was similar across the PCs (*p* < 0.05). At the end of PC‐4, the *p* values of the three substrates were within the optimal range described by Bunt (1988).

#### 3.2.5 Air volume at −10 cm

Figure 1e shows significant differences in AV for both factors analyzed (*p* < 0.05). With respect to substrate, the control had the greatest percentage of AV in PC‐0 and PC‐1 (32%–34%), which is also related to the higher percentage of CF. CF has been reported to present an AV of 10%–15% (Meerow, 1994). In contrast, 60V40CF presented the lowest percentage of AV, which was inversely related to the high BD in this substrate. These results show the direct relationships between OM and AV and between P and AV, as well as the inverse relation between BD and AV. For the different PCs, a significant reduction (*p* < 0.05) in AV was observed from PC‐2 onward in the three substrate treatments because of the formation and greater presence of smaller particle sizes. As the OM is processed by microorganisms, such small particles directly influence the porous space and BD. According to Raviv, Zaidman, and Kapulnik (1998), the size, shape, and distribution of particles influence the water and air availability in the substrates. The range of AV values was above 10%, which is the minimum level described by Abad et al. (2004).

#### 3.2.6 Readily available water

Readily available water values showed significant differences for the two factors analyzed (*p* < 0.05) (Figure 1f). With respect to substrate, at the end of PC‐4, nearly similar results were obtained for 40V60CF (32.7%) and 60V40CF (32.3%). In contrast, the control presented a smaller percentage (25%) than the recommended optimal amount (>30%) (*p* < 0.05). In the case of the PC effect, RAW increased for the 40V60CF and 60V40CF substrates with successive PCs, while it decreased in the control treatment. The RAW values in 40V60CF and 60V40CF were within the optimal range (>30%) according to Abad et al. (2004). In particular, RAW is an important physical characteristic of organic substrates used for horticultural production from a productive and economic perspective, as RAW is one of the characteristics that determines reuse potential.

The reduction in RAW in the control treatment is explained by the tendency of CF to form particles of small diameter (coir dust), coinciding with the assertions of Bunt (1988), who stated that high or low P in a substrate depends on the size distribution of the pores: large pores in mulch trigger water loss by gravity, and with very small pores, the matric potential is higher than −50 cm. Therefore, the energy or negative pressure with which water is retained in the soil is also higher; the plant is unable to extract much of the water before wilting because the water is less available to the plant (reduction of RAW) (Bunt, 1988). This also explains the inverse relation between OM and RAW, which is due to the greater presence of macropores (large pores) in OM, leading to lower water retention and, as a consequence, lower RAW.

According to the results, we suggest that the control treatment exhibits changes in its structure from PC‐3 onward after the quantity of macropores has been reduced and more small pores (dust) have been produced because of the high CF content in this treatment. Accordingly, a reduction in RAW occurs, and re‐use of the control may be ruled out.

#### 3.2.7 Pearson product moment correlations among the physical characteristics of the substrates

**Figure 2:**
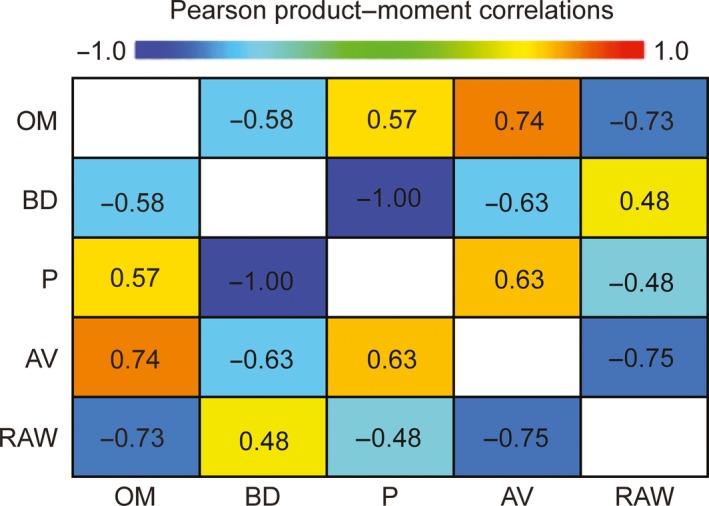
Pearson product‐moment correlations between physical properties

The strength of the previously explained positive (direct) and negative (inverse) relations are shown in Figure 2. The following positive (direct) relations are noted in the order of the greatest to the smallest correlation: OM–AV (0.74), P–AV (0.63), and OM–P (0.57). In addition, the following negative (inverse) correlations are noted: AV–RAW (−0.75), OM–RAW (−0.73), BD–AV (−0.63), and OM–BD (−0.58).

### 3.3 Chemical properties of the substrates

**Figure 3:**
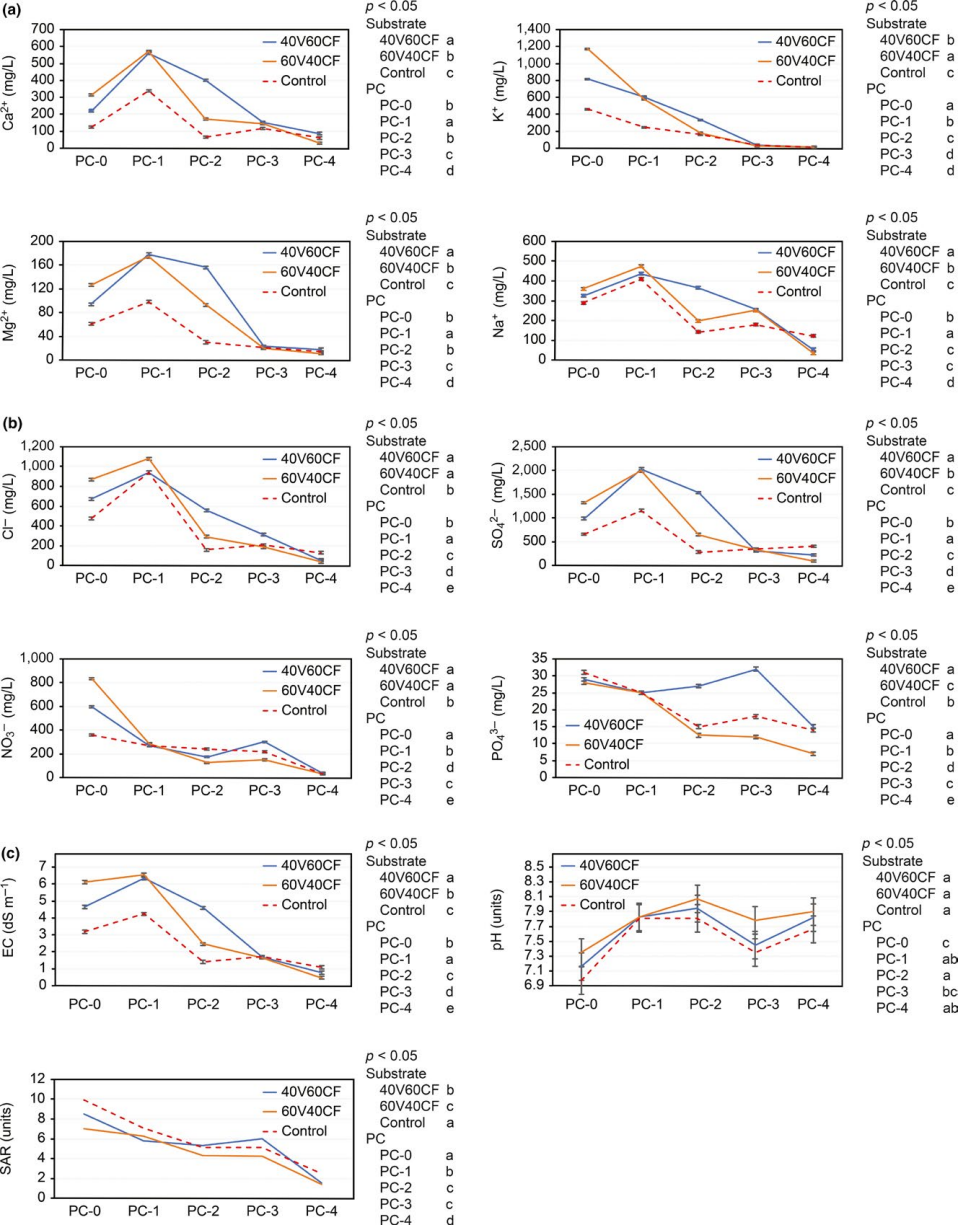
(a) SME cations (Ca^2+^, K^+^, Mg^2+^, Na^+^) between substrate treatments and production cycles. (b) SME anions (Cl^−^, SO_4_^2−^, NO_3_^−^, PO_4_^3−^) between substrate treatments and production cycles. (c) SME electrical conductivity (EC), pH, and sodium adsorption ratio (SAR) between substrate treatments and production cycles. Different letters in the substrate legends indicate *p* < 0.05 between substrate treatments. Different letters in the PC legends indicate *p* < 0.05 between production cycles (*n* = 3). Error bars, Fisher's LSD test (95%)

In Figure 3a–c, all interrelations with respect to the analyses of the SME cations and anions, EC, SAR, and pH are presented with consideration of the substrate and PC factors. In general, a clear tendency (*p* < 0.05) exists for the concentration of ions to decrease over the course of the PCs. At the end of PC‐4, the ion levels are below the required optimal levels for acceptable production. The results demonstrate that the substrates with the highest percentage of V (40V60CF, 60V40CF) also present the highest levels of cations and anions in comparison to the control. Considering pH, there were no significant differences between substrates, but there were differences (*p* < 0.05) among PCs. We suggest that there could be a greater exchange of anions as the different crops were planted because the roots of the plants use OH− radicals to exchange anions, which reduces the amount of H+ cations (reducing the cation exchange capacity). This is also related to the high level of sulfates, phosphates and chlorides present in the substrate (vermicompost).

According to the classification of IW in the USDA Handbook No. 60 (Richards, 1954), at the end of PC‐4, the SAR and EC of the substrates classify the substrates within a range of C2–S1, which corresponds to average salinity (C2) and low sodium content (S1). Therefore, these substrates could be used for the production of crops with moderate salinity tolerance (Richards, 1954).

### 3.4 Biological properties of the substrates

#### 3.4.1 Dehydrogenase

**Figure 4:**
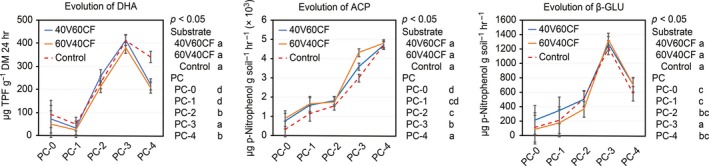
Enzymatic activity between substrate treatments and production cycles. (a) DHA; (b) ACP; (c) β‐GLU. Different letters in the substrate legends indicate *p* < 0.05 between substrate treatments. Different letters under the PC legends indicate *p* < 0.05 between production cycles (replicates *n* = 3). Error bars, Fisher's LSD test (95%)

**Figure 5:**
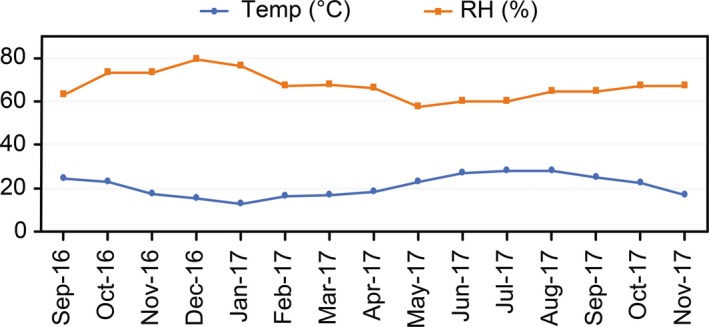
Temperature (°C) and relative humidity (%) inside the greenhouse (average daily temperature)

Figure 4a shows the results of DHA activity (μg TFF g^−1^ DW 24 hr) based on 10 evaluations during the four PCs. With respect to substrate, no significant differences were found (*p* < 0.05). However, a significant difference was found with respect to PC. A small decrease in DHA activity was observed at the end of PC‐1 (<60 μg TFF g^−1^ DW 24 hr). Subsequently, DHA activity significantly increased (*p* < 0.05) in PC‐2 and reached its maximum in PC‐3 (350–400 μg TFF g^−1^). We suggest that the decrease in PC‐1 could be due to an initial adaptation phase of microbiota before reaching the maximum growth phase that led to increased activity. Therefore, in PC‐2 and PC‐3, the DHA activity achieved its maximum before decreasing in PC‐4 (*p* < 0.05), likely as a result of high temperatures (Figure 5) during summer (the transition to summer occurred before PC‐4 initiated) and lower nutrient availability. Accordingly, a reduction in the microbiota occurred, which led to reduced enzymatic activity. This issue has also been reported by Biederbeck, Zentner, and Campbell (2005). The results also demonstrate that the use of continuous planting cycles (PC‐2, PC‐3) favors an increase in DHA activity, which in turn reflects the improved growth potential of microbiota in the rhizosphere, as reported by Vargas‐García, Suárez‐Estrella, López, and Moreno (2010). The values for the DHA activity in the substrates evaluated in this study are greater than those reported by Bhat et al. (2017) for soils under organic production.

#### 3.4.2 Acid phosphatase

Figure 4b presents the results of ACP activity (μg p‐nitrophenol g soil^−1^ hr^−1^) (10 evaluations). The values of this enzyme did not show significant differences among substrates (*p* < 0.05). With respect to PC, ACP tended to increase (*p* < 0.05) over the course of the PCs. The ACP values were low (<1,000 μg p‐nitrophenol g^−1^ soil hr^−1^) in PC‐1 and increased afterward, reaching the highest values by PC‐4 (4,700 μg p‐nitrophenol g^−1^ soil hr^−1^). The arrival of summer (high temperature and low humidity) before PC‐4 began did not lead to a reduction in ACP in the evaluated substrates. We suggest that this issue is related to the findings of Margalef et al. (2017), who stated that phosphatase enzymes are produced by bacteria, fungi and plant roots and serve to cleave a phosphate group from its substrates, transforming complex and sometimes unavailable forms of organic phosphorus into assimilable phosphate. Therefore, soil contains large quantities of intracellular (in living microbial cells) and extracellular (secretions of living cells or dead cellular material) phosphatases. Furthermore, phosphatases can be stabilized in the soil on surface‐reactive particles (e.g., clay and iron or aluminum oxides). This geochemically immobilized and yet enzymatically active fraction accounts for the enzymatic activity exhibited by soil, even in the absence of living organisms.

The values of the ACP activity found in this study are within the optimal ranges of phosphatase activity published by Bhat et al. (2017).

#### 3.4.3 β*‐*glucosidase

Figure 4c presents the results of β‐GLU activity (10 evaluations). Similar to the other enzymes analyzed, β‐GLU activity did not show significant differences among substrates (*p* < 0.05). With respect to PC, β‐GLU activity had an initial average value of 100–200 μg p‐nitrophenol g^−1^ soil hr^−1^ and then significantly increased (*p* < 0.05) in PC‐3 to an average of 1,200 (μg p‐nitrophenol g^−1^ soil hr^−1^). Afterward, β‐GLU activity decreased (*p* < 0.05) (600 μg p‐nitrophenol g^−1^ soil hr^−1^) during PC‐4. The increase in β‐GLU activity in PC‐3 could be related to improved OM degradation activity by microbiota, which occurs during continuous PCs fueled by exudates in the rhizosphere. Essentially, over time, the microbiota reached their maximum growth potential. A reduction in the β‐GLU activity was observed in the final PC (PC‐4), likely due to climatic factors (the arrival of summer) along with the depletion of simple energy sources (glucose monomers) that limited the growth of microorganisms. For this reason, β‐GLU was used as a sensitive indicator of microbially mediated soil processes, as reported by Stott, Andrews, Liebig, Wienhold, and Karlen (2010). In particular, β‐GLU is involved in the decomposition of cellulose compounds and is synthesized by soil microorganisms in the presence of suitable substrates. Furthermore, Cunha‐Queda, Ribeiro, Ramos, and Cabral (2007) reported that low activity of β‐GLU in the final stages of composting is one consequence of the decrease in available organic substrates. The values of β‐GLU activity in this study were greater than those reported by several previously cited authors for soil under organic management (Bhat et al., 2017; Cordovil et al., 2017; de Almeida et al., 2015).

### 3.5 PCA of physical properties and enzymatic activity (DHA, ACP, β‐GLU)

**Figure 6:**
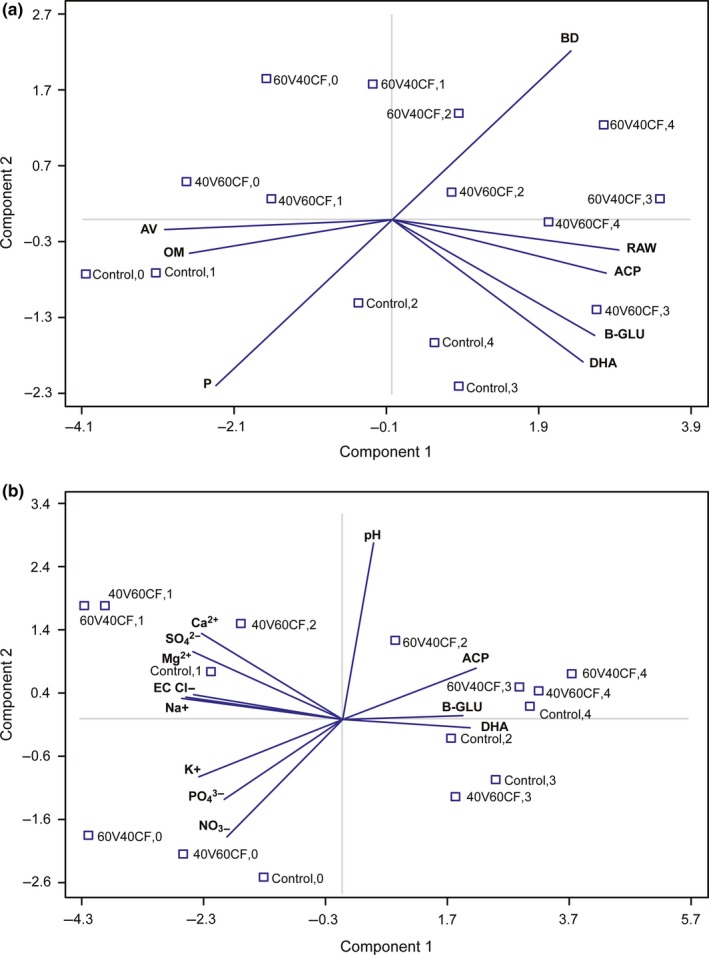
Principal components analysis (PCA) between physicochemical and biological parameters. (a) Bi‐plot of component weights between physical properties (OM, BD, P, AV, RAW) and enzymatic activities (DHA, ACP, β‐GLU): first component, 64.98% of the variance; second component, 19.38% of the variance. (b) Bi‐plot of component weights between SME and enzymatic activities (DHA, ACP, β‐GLU): first component, 67.67% of the variance; second component, 15.80% of the variance

PCA was performed to describe the factors that explain most of the variability in the analyzed parameters and to identify the physicochemical parameters and enzyme activities that could work synergistically between them. To select the principal components to extract, all components were considered, and those with an eigenvalue greater than 1 were selected (fraction 1/p of the total population variance). Figure 6a shows the PCA gathering the physical properties and enzymatic activity. Two principal components described 84.36% of the total variation. The variables of component 1 showed a direct correlation and synergism among the RAW, DHA, ACP, and β‐GLU. This model coincides with that reported by Miras‐Avalos, Fouz, and Vázquez (2007), who found a marked parallelism between the activity of DHA and moisture content in the soil. The relation between the ACP and RAW has been reported by Margalef et al. (2017), who suggested that total nitrogen, mean annual precipitation, mean annual temperature, and total soil carbon were the most available predictor variables, explaining up to 50% of the spatial variance in the phosphatase activity. Component 2 expressed the correlation or synergism between AV and OM and highlighted the previously described inverse relation between BD and P. Figure 6a also shows a relationship between OM and the treatments (between control and PC‐0 and between control and PC‐1) with a higher content of OM observed at the beginning of the study. There was also a negative correlation between OM and enzymatic activity (DHA, ACP, β‐GLU). A higher content of OM does not always imply immediately higher enzymatic activity because the start of a crop in organic substrate involves the process of stabilization and transformation of OM (mineralization). This in turn is very dependent on the microbial lag phase, which is the adaptation of the indigenous microbiota to new cultivation and environmental conditions. Once this adaptation process is completed, the exponential growth phase begins, which involves a greater microbial and enzymatic activity in the mineralization processes. This issue was reported by Swinnen, Bernaerts, Dens, Geeraerd, and Van Impe (2004). Therefore, during the crop cycles (PC‐O, PC‐1, PC‐2, PC‐3, PC‐4), OM will be reduced and transformed to an assimilable compound (mineralization) for plants, while enzymatic activity will increase in the exponential phase of growth of the indigenous microbiota and the rhizospheric zone. Thus, OM decreases, and enzymatic activity increases during the PCs (from PC‐0 to PC‐4).

Figure 6a also shows that the enzymatic activity, RAW, and BD were directly linked with PC‐2, PC‐3, and PC‐4. We suggest that this proves that the rhizosphere microbiota reached their greatest growth potential during these production cycles and achieved the highest transformation of organic sources, thereby leading to increases in BD and RAW (Figure 1c,f).

The consecutive production cycles left root residues that provided OM, enabling the improved growth of microorganisms. Once mineralized by microorganisms, these crop residues represent a source of nutrients for subsequent crops.

The results of the PCA between chemical properties (SME) and enzymatic activity are presented in Figure 6b. Three principal components with eigenvalues greater than 1 were identified. Components 1 and 2 explained 83.47% of the total variance. The greatest concentration of cations and anions is linked to the initial PCs of PC‐0 and PC‐1. The greatest variability was associated with six variables divided into two groups that exhibited strong synergistic relations. The first group was formed by EC, Cl^−^, and Na^+^, whereas the second group was formed by SO_4_^2−^, Ca^2+^, and Mg^2+^. Additionally, the essential nutrients K, P, and N are shown to exist in the substrate before cultivation (PC‐0). The contribution of these nutrients via exogeneous sources is indispensable, as these nutrients promote crop yield and contribute to the re‐use potential of substrates.

Figure 6b also showed that pH was not correlated with enzymatic activity, and extremely high or low pH values generally result in complete loss of activity for most enzymes. pH is also a factor that affects the stability of enzymes. For each enzyme, there is also a range of pH values for optimal stability. The optimum pH value will vary greatly from one enzyme to another. DHA has an optimum pH of 7.2 (Aquino Neto, Forti, Zucolotto, Ciancaglini, & De Andrade, 2011), ACP of 4.8–5.8 (Mobley, Chengappa, Kadel, & Stuart, 1984) and β‐GLU of 6.6 (Assoi Yapi, Gnakri, Niamke, & Kouame, 2009). In this research, the pH was in the range of 7.0–8.0, and there was no clear relationship or trend between pH and enzymatic activity. We suggest that the pH was in a suitable range for enzymatic activity; therefore, it was not an influential factor in the results.

With respect to enzymatic activity, Figure 6b shows the synergism of DHA, ACP, and β‐GLU with PC‐2, PC‐3, and PC‐4, which is related to the greater activity of microbiota in successive crops.

## 4 CONCLUSIONS

The physical characteristics of the evaluated substrates after four PCs indicate that the control substrate (20V80CF) did not comply with the established optimal RAW values for continued production (reuse). The substrate 60V40CF was also ruled out for continued production, as the measured BD values exceeded the maximum acceptable value.

According to the chemical characteristics of the substrates after four PCs, the minimum levels of cations and anions were found. However, salinity or alkalinity problems were not observed. The substrate 40V60CF had a higher concentration of nutrients in SME.

The biological characteristics indicated that the evaluated substrates (V and CF) presented high enzymatic activity (DHA, ACP, β‐GLU), which guaranteed the transformation and assimilation of nutrients and elements in inorganic form as well as the availability of minerals for the production of four crops.

According to the results in this research, we suggest that the 40V60CF substrate mixture is the most adequate for organic production over the course of four production cycles. This mixture maintained the physical properties of soil at the recommended levels and exhibited high enzymatic activity and greater cation and anion concentrations over the course of the PCs. For these reasons, this mixture is a good candidate for re‐use when supplemented with organic nutrients or minerals.

## CONFLICT OF INTEREST

None declared.

## ETHICAL STATEMENT

All authors declare that have read the Editorial Policies, where stated that this research was carried out in the presence of any personal, professional, or financial relationships that cannot be construed as a conflict of interests.
