# Supplementary material for: Evaluation of physicochemical properties and enzymatic activity of organic substrates during four crop cycles in soilless containers
Source: Food Sci Nutr. 2018 Oct 19;6(8):2066–78. doi: 10.1002/fsn3.757 (PMC6261231; doi:10.1002/fsn3.757)
Supplement: Supplementary file 1 [file FSN3-6-2066-s001.pdf]

Spanish standard

UNE-EN 13040

April 2008

Soil improvers and growing media

Sample preparation for chemical and physical tests

Determination of dry matter content, moisture content and laboratory compacted bulk density

This standard has been prepared by the Technical Committee CTN 142 *Fertilizers, liming materials and means of growing* the Secretariat of which is held by ANFFE.

Asociación Española
de Normalización
Génova, 6 - 28004 Madrid
915 294 900
info@une.org
www.une.org

Este documento ha sido adquirido por Pedro Antonio Mejia Guerra el 2018-7-22.
Para poder utilizarlo en un sistema de red interno, deberá disponer de la correspodiente licencia de AENOR

UNE-EN 13040

Soil improvers and growing media
Sample preparation for chemical and physical tests
Determination of dry matter content, moisture content and laboratory compacted bulk density

Mejoradores de suelo y sustratos de cultivo. Preparación de la muestra para ensayos físicos y químicos. Determinación del contenido de materia seca, del contenido de humedad y de la densidad aparente compactada en laboratorio.

Amendements organiques et supports de culture. Préparation des échantillons pour les essais physiques et chimiques, détermination de la teneur en matière sèche, du taux d'humidité et de la masse volumique compactée en laboratoire.

This standard is the official English version of EN 13040:2007.

This standard was published as UNE-EN 13040:2008, which is the definitive Spanish version.

The remarks to this document must be sent to:

Asociación Española de Normalización

Génova, 6
28004 MADRID-España
Tel.: 915 294 900
info@une.org
www.une.org
Depósito legal: M 13957:2018

© UNE 2018
Published by AENOR INTERNACIONAL S.A.U. under licence of Asociación Española de Normalización.
Forbidden reproduction

Asociación Española de Normalización, UNE

Este documento ha sido adquirido por Pedro Antonio Mejia Guerra el 2018-7-22.
Para poder utilizarlo en un sistema de red interno, deberá disponer de la correspodiente licencia de AENOR

EUROPEAN STANDARD

NORME EUROPÉENNE

EUROPÄISCHE NORM

EN 13040

October 2007

ICS 65.080

Supersedes EN 13040:1999

English Version

Soil improvers and growing media - Sample preparation for chemical and physical tests, determination of dry matter content, moisture content and laboratory compacted bulk density

Amendements organiques et supports de culture - Préparation des échantillons pour les essais physiques et chimiques, détermination de la teneur en matière sèche, du taux d'humidité et de la masse volumique compactée en laboratoire

Bodenverbesserungsmittel und Kultursubstrate - Probenherstellung für chemische und physikalische Untersuchungen, Bestimmung des Trockenrückstands, des Feuchtigkeitsgehaltes und der Laborschüttdichte

This European Standard was approved by CEN on 26 August 2007.

CEN members are bound to comply with the CEN/CENELEC Internal Regulations which stipulate the conditions for giving this European Standard the status of a national standard without any alteration. Up-to-date lists and bibliographical references concerning such national standards may be obtained on application to the CEN Management Centre or to any CEN member.

This European Standard exists in three official versions (English, French, German). A version in any other language made by translation under the responsibility of a CEN member into its own language and notified to the CEN Management Centre has the same status as the official versions.

CEN members are the national standards bodies of Austria, Belgium, Bulgaria, Cyprus, Czech Republic, Denmark, Estonia, Finland, France, Germany, Greece, Hungary, Iceland, Ireland, Italy, Latvia, Lithuania, Luxembourg, Malta, Netherlands, Norway, Poland, Portugal, Romania, Slovakia, Slovenia, Spain, Sweden, Switzerland and United Kingdom.

EUROPEAN COMMITTEE FOR STANDARDIZATION
COMITÉ EUROPÉEN DE NORMALISATION
EUROPÄISCHES KOMITEE FÜR NORMUNG

Management Centre: rue de Stassart, 36 B-1050 Brussels

© 2007 CEN All rights of exploitation in any form and by any means reserved worldwide for CEN national Members.

Ref. No. EN 13040:2007: E

Asociación Española de Normalización, UNE

Este documento ha sido adquirido por Pedro Antonio Mejia Guerra el 2018-7-22.
Para poder utilizarlo en un sistema de red interno, deberá disponer de la correspodiente licencia de AENOR

Contents

Page

Foreword ... 3

1 Scope ... 4

2 Normative references ... 4

3 Terms and definitions ... 4

4 Principle ... 4

5 Sampling ... 5

6 Sample reception ... 5

7 Transportation and storage of samples ... 5

8 Preparation of the un-dried test sample ... 5
8.1 Sample preparation ... 5
8.2 Determination of material exceeding 40 mm ... 6
8.3 Test sample passing through a 40 mm square aperture sieve ... 6
8.4 Test sample passing through a 25 mm square aperture sieve ... 6
8.5 Test sample passing through a 20 mm square aperture sieve ... 6

9 Preparation of the dried ground (or otherwise size reduced) test sample ... 7
9.1 Apparatus ... 7
9.2 Procedure ... 7

10 Determination of dry matter content ... 7
10.1 Apparatus ... 7
10.2 Procedure ... 7

11 Calculation ... 8
11.1 Dry matter content ... 8
11.2 Moisture content ... 8

12 Precision - Moisture content ... 8

13 Test report ... 8

Annex A (normative) Determination of laboratory compacted bulk density ... 10
A.1 General ... 10
A.2 Principle ... 10
A.3 Apparatus ... 10
A.4 Procedure ... 12
A.5 Expression of results ... 13
A.6 Use and storage of material ... 14
A.7 Precision - Compacted laboratory bulk density ... 14
A.8 Test report ... 14

Annex B (informative) Results of an interlaboratory trial to determine moisture content and laboratory compacted bulk density ... 15

Bibliography ... 17

Asociación Española de Normalización, UNE

Este documento ha sido adquirido por Pedro Antonio Mejia Guerra el 2018-7-22.
Para poder utilizarlo en un sistema de red interno, deberá disponer de la correspodiente licencia de AENOR

Foreword

This document (EN 13040:2007) has been prepared by Technical Committee CEN/TC 223 “Soil improvers and growing media”, the secretariat of which is held by BSI.

This European Standard shall be given the status of a national standard, either by publication of an identical text or by endorsement, at the latest by April 2008, and conflicting national standards shall be withdrawn at the latest by April 2008.

Attention is drawn to the possibility that some of the elements of this document may be the subject of patent rights. CEN [and/or CENELEC] shall not be held responsible for identifying any or all such patent rights.

This document supersedes EN 13040:1999.

According to the CEN/CENELEC Internal Regulations, the national standards organizations of the following countries are bound to implement this European Standard: Austria, Belgium, Bulgaria, Cyprus, Czech Republic, Denmark, Estonia, Finland, France, Germany, Greece, Hungary, Iceland, Ireland, Italy, Latvia, Lithuania, Luxembourg, Malta, Netherlands, Norway, Poland, Portugal, Romania, Slovakia, Slovenia, Spain, Sweden, Switzerland and the United Kingdom.

Safety warning

Take care when handling samples that may contain sharps or are of a dusty nature. Samples should be handled with latex gloves and in the case of dusty materials with mask and gloves.

Asociación Española de Normalización, UNE

Este documento ha sido adquirido por Pedro Antonio Mejia Guerra el 2018-7-22.
Para poder utilizarlo en un sistema de red interno, deberá disponer de la correspodiente licencia de AENOR

1 Scope

This European Standard specifies a routine method for preparing a sample of soil improver or growing media prior to chemical analysis and physical testing. The procedures described herein apply only to those samples that are supplied to a laboratory in the form in which they will be used for their intended purpose.

NOTE 1 This method is not applicable to liming materials and is not suitable for materials like rockwool and foam slabs.

NOTE 2 The determination of the laboratory compacted bulk density is given in Annex A.

NOTE 3 The results of an interlaboratory trial to determine moisture content are given in Annex B.

NOTE 4 The results of an interlaboratory trial to determine compacted laboratory bulk density are given in Annex B.

NOTE 5 Attention is drawn to the possible existence of national legislation for the declaration of specific products, which could differ from the general requirements of this European Standard.

2 Normative references

The following reference documents are indispensable for the application of this European Standard. For dated references, only the edition cited applies. For undated references, the latest edition of the referenced document (including any amendments) applies.

EN 12579:1999, *Soil improvers and growing media — Sampling*

ISO 565, *Test sieves — Metal wire cloth, perforated metal plate and electroformed sheet — Nominal sizes of openings*

3 Terms and definitions

For the purposes of this standard, the terms and definitions in EN 12579:1999 and the following apply.

3.1
test sample
sample prepared from the laboratory sample and from which test portions will be taken

3.2
test portion
quantity of material drawn from the test sample (or from the laboratory sample if both are the same) and on which the tests or observations are actually carried out

3.3
laboratory compacted bulk density
density, expressed in grams per litre of the material as determined in the laboratory using a 1 l cylinder; the sample being compacted under defined conditions

4 Principle

The laboratory sample is coded and sub-divided to prepare the test sample in such a manner as to be representative of the sample as submitted to the laboratory. The sample's intrinsic structure shall be maintained whenever possible.

Este documento ha sido adquirido por Pedro Antonio Mejia Guerra el 2018-7-22.
Para poder utilizarlo en un sistema de red interno, deberá disponer de la correspodiente licencia de AENOR

Asociación Española de Normalización, UNE

5 Sampling

The laboratory sample shall be obtained in accordance with EN 12579.

6 Sample reception

Upon receipt of the laboratory sample, the laboratory shall confirm that the sample relates to the accompanying documentation. The sampler shall submit with the sample, at least the following minimum requirements:

a) name of the client;

b) to whom the results shall be reported if different from a) above;

c) place and date the sample was taken;

d) name of the sampler;

e) discrete sample identification; and

f) analysis required.

The laboratory shall confirm that a sufficient sample has been provided for the analyses to be undertaken by recording the date the sample was received and giving the sample a unique laboratory identification code. This code shall be recorded on all subsequent sub sample containers and on the documentation supplied with the sample. Analysis shall be undertaken within 2 weeks of receipt of the sample.

7 Transportation and storage of samples

The laboratory sample shall be transported and stored without compaction or any other treatment which may irreversibly alter its moisture content, particle size, packing characteristics or any feature which affects density.

A sub-sample or sample of not less than 5 l, as submitted to the laboratory shall be stored so that it shall not undergo any further decomposition, physical damage, hydration or dehydration. Recommended storage should be in a closed polyethylene bag so that the sample fills the container with no free air at 1 °C to 5 °C, but not frozen. The storage period depends on several factors including what is the normal custom in the analysing laboratory or country. It is recommended that all such samples should be stored for a minimum of 28 days from the date of reporting the results to the client. The expected storage period shall be reported to the client at the time the results are reported.

8 Preparation of the un-dried test sample

8.1 Sample preparation

Thoroughly mix the laboratory sample, gently breaking any lump or agglomerate of the sample that has been caused, by, for example, compression during transportation.

NOTE Care should be taken to avoid breaking intrinsic parts and to avoid a loss of moisture.

If necessary, divide the sample to form sub-samples. Recognized procedures such as coning and quartering shall be used for sub-sampling. The procedure used shall be included in the report. The size of the final test sample shall be large enough to truly represent the laboratory sample and to

Asociación Española de Normalización, UNE

Este documento ha sido adquirido por Pedro Antonio Mejia Guerra el 2018-7-22.
Para poder utilizarlo en un sistema de red interno, deberá disponer de la correspodiente licencia de AENOR

provide sufficient uniform material for all defined physical and chemical tests that are required to be carried out. It is unlikely that a laboratory sample of less than 10 l shall be sufficient for all physical and chemical analyses.

During preparation the sample shall not be cut or ground.

8.2 Determination of material exceeding 40 mm

Weigh approximately 1 000 ml of the test sample (m_a) and pass it through a 40 mm square aperture sieve and agitate gently if required.

Record the weight (m_b) of the amount of sample that does not pass the sieve and express this figure (c) as a fraction of the total sub-sample mass. This figure is to be reported.

$$c = \frac{m_b}{m_a} \qquad (1)$$

where

m_a is the mass in grams of the sub-sample;

m_b is the mass in grams of material retained on a 40 mm square aperture sieve;

c is the fraction retained on a 40 mm square aperture sieve.

8.3 Test sample passing through a 40 mm square aperture sieve

When 20 % w/w or less of the laboratory sample (8.1) has been retained on the 40 mm sieve, the retained particles shall be physically reduced in equal parts as few times as necessary to permit the entire sample to pass through the sieve.

Thoroughly mix the whole sub-sample with the broken particles that have been retained on the sieve taking care to minimize physical damage to the sample as a whole. Any observed foreign material such as plastic, metal or glass shall be recorded. Include this observation in the test report (13).

8.4 Test sample passing through a 25 mm square aperture sieve

Take approximately 10 l of the test sample (8.1) and pass through a 25 mm sieve. Any particle of the sample > 25 mm and /or flexible fibres > 80 mm shall be physically reduced in equal parts and as few times as are necessary to be ≤ 25 mm and ≤ 80 mm for flexible fibres.

Thoroughly mix the whole sub-sample with the broken particles that have been retained on the sieve taking care to minimize physical damage to the sample as a whole. Any observed foreign material such as plastic, metal or glass shall be recorded. Include this observation in the test report (13).

NOTE This test sample is suitable for physical methods of analyses.

8.5 Test sample passing through a 20 mm square aperture sieve

Take about 5 l of test sample (8.3) and using a scoop, pass the material through a 20 mm screen and agitate gently if required. If more than 10 % volume is retained on the screen then the procedure shall be inappropriate to the material under test. If less than 10 % is retained, this material shall be broken down in equal parts and as few times as necessary to permit the entire sample to pass through the sieve.

Asociación Española de Normalización, UNE

Este documento ha sido adquirido por Pedro Antonio Mejia Guerra el 2018-7-22.
Para poder utilizarlo en un sistema de red interno, deberá disponer de la correspodiente licencia de AENOR

9 Preparation of the dried ground (or otherwise size reduced) test sample

9.1 Apparatus

9.1.1 Grinding apparatus, able to grind the whole sub-sample without contamination, e.g. cutting mill, ultracentrifuge mill, pestle and mortar.

9.1.2 Screen or sieve, of diameter 2 mm round hole in accordance with ISO 565.

9.1.3 Ventilated oven, capable of maintaining a temperature of 75 °C ± 5 °C or other means of sample drying

9.2 Procedure

Dry a portion of the test sample (8.1) until it crumbles to the touch, using one of the following methods:

a) at 75 °C ± 5 °C in a ventilated oven; or

b) where it is necessary to prevent losses by conventional oven drying methods, freeze drying or milling in the presence of dry ice.

It shall be recorded with the results when a technique like the type presented in b) is used.

The particle size shall be reduced so that the dried sample is able to pass through the 2 mm mesh sieve (9.1.2). It may be necessary to chop, cut or otherwise reduce large particles prior to milling. For samples that can be milled, ensure that during grinding no heat is generated and no inadvertent sub-sampling occurs, in that some particle sizes are excluded from the milling process either as dust or as excessively hard particles. For samples that cannot be milled, e.g. expanded foam, other means for reducing the particulate size such as knives or scissors may be used.

10 Determination of dry matter content

10.1 Apparatus

10.1.1 Sample tray, capable of holding no less than 50 g of the sample and constructed of material thermally stable up to 150 °C.

10.1.2 Drying oven, ventilated, fan assisted, capable of holding sample trays (10.1.1) and maintaining 103 °C ± 2 °C.

10.1.3 Analytical balance, with a scale interval of 0,01 g and a capacity of weighing 500 g.

10.2 Procedure

Determine the mass of the empty tray (m_T), by heating it to 103 °C in an oven and cool it in the desiccator. After cooling weigh the tray to get (m_T). Transfer approximately 50 g of the mixed prepared sample (8.1) in the tray (10.1.1), spread to an even depth not exceeding 2 cm. and without delay weigh to an accuracy of 0,01 g. (m_W). Place the tray in the oven (10.1.2) and dry until the difference between two successive weighings does not exceed 0,1 g. Record the dry mass of the sample and tray (m_D).

NOTE Loss of volatile matter — Drying the sample at 75 °C and 103 °C may lead to losses of certain volatile components such as free ammonia. Therefore, where these components are to be determined the analyses shall be performed on the un-dried sample.

Este documento ha sido adquirido por Pedro Antonio Mejia Guerra el 2018-7-22.
Para poder utilizarlo en un sistema de red interno, deberá disponer de la correspodiente licencia de AENOR

Asociación Española de Normalización, UNE

11 Calculation

11.1 Dry matter content

Calculate the dry matter content of the sample, as received, using the following equation:

$$D_M = \frac{(m_D - m_T)}{(m_W - m_T)} \times 100 \quad (2)$$

where

D_M is the dry matter content expressed as a percentage by mass;

m_W is the mass in grams of the wet sample plus tray;

m_D is the mass in grams of the dried sample plus tray;

m_T is the mass in grams of the empty dry tray.

11.2 Moisture content

Calculate the moisture content of the sample as received using the following equation:

$$W_m = \frac{(m_W - m_D)}{(m_W - m_T)} \times 100 \quad (3)$$

where

W_m is the moisture content expressed as a percentage by mass;

m_W is the mass in grams of the wet sample plus tray;

m_D is the mass in grams of the dried sample plus tray;

m_T is the mass in grams of the empty dry tray.

12 Precision - Moisture content

The precision of the moisture content measurement of three separately prepared samples should be in accordance with Table B.1.

A summary of the results of an interlaboratory trial to determine the precision of the method in accordance with ISO 5725 [1] to [6] is given in Annex B.

NOTE The values derived from the interlaboratory trial may not be applicable to concentrations and matrices other than those given.

13 Test report

The test report shall contain the following information:

a) reference to this European Standard (EN 13040:2007);

Este documento ha sido adquirido por Pedro Antonio Mejia Guerra el 2018-7-22.
Para poder utilizarlo en un sistema de red interno, deberá disponer de la correspodiente licencia de AENOR

Asociación Española de Normalización, UNE

b) all information necessary for complete identification of the sample;

c) percentage by mass of material that has been cut according to clause 8.2;

d) method and temperature of drying;

e) results of the determination to the nearest 0,1 % by mass;

f) details of any operations not specified in the European Standard or regarded as optional, as well as any factor which may have affected the results.

Asociación Española de Normalización, UNE

Este documento ha sido adquirido por Pedro Antonio Mejia Guerra el 2018-7-22.
Para poder utilizarlo en un sistema de red interno, deberá disponer de la correspodiente licencia de AENOR

Annex A
(normative)

Determination of laboratory compacted bulk density

A.1 General

A.1.1 This annex specifies a method to estimate the laboratory compacted bulk density of a laboratory sample of a soil improver or growing media in its "as received state" and enables a calculated sample weight to be taken, which will accurately represent the required (small) volume of sub-sample as required for analysis.

A.1.2 This method enables computation and expression of analytical results on a weight in volume basis when results are obtained from analysis of a known weight of sub-sample.

NOTE This procedure may be inappropriate for use with wet, sticky or very coarse materials.

A.2 Principle

Fit a one litre cylinder with an extension collar, fixed screen/flow beaker and funnel, and fill it with the material. The fixed screen/flow breaker shall be omitted for materials that contain more than 10 % by weight greater than 20 mm in any dimension. Apply compaction, except for very compressible or springy materials, and weigh the final contents of the cylinder.

A.3 Apparatus

A.3.1 Test Cylinder

A rigid test cylinder as shown in Figure A.1, having a nominal capacity of 1 000 ml ± 30 ml, and a diameter (d) of 99 mm - 105 mm in accordance with Table A.1.

Asociación Española de Normalización, UNE

Este documento ha sido adquirido por Pedro Antonio Mejia Guerra el 2018-7-22.
Para poder utilizarlo en un sistema de red interno, deberá disponer de la correspodiente licencia de AENOR

Dimensions in millimetres

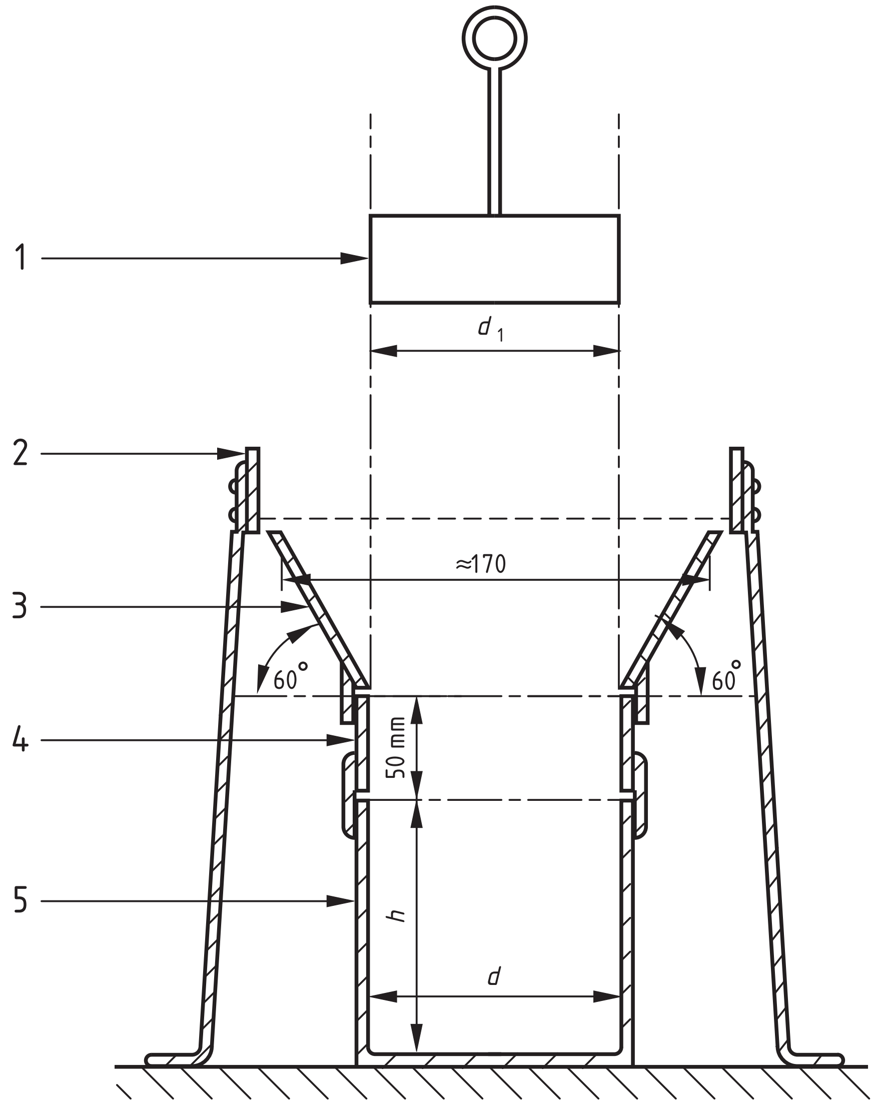

Key

1 plunger (for mass see Table A.1)
2 supported fall controller
3 funnel
4 removable collar
5 test cylinder
d diameter as per Table A.1
h height as per Table A.1
d_1 diameter (d - 5mm) ± 1 mm

Figure A.1 — Laboratory compacted bulk density test cylinder, collar, fall controller and plunger

Asociación Española de Normalización, UNE
Este documento ha sido adquirido por Pedro Antonio Mejia Guerra el 2018-7-22.
Para poder utilizarlo en un sistema de red interno, deberá disponer de la correspodiente licencia de AENOR

Table A.1 — Dimensions of cylinder and corresponding plunger mass

Diameter (d) (mm)	Height (h) (mm)	Volume (ml)	Mass of Plunger (g)
**99,0**	130	1 000	634
**100,0**	127	997	650
**101,0**	125	1 001	664
**102,0**	122	997	678
**103,0**	120	1 000	692
**104,0**	118	1 002	706
**105,0**	116	1 004	720
**Tolerance**	± 1mm	± 30ml	± 5g

The capacity should be checked by weighing to the nearest 1 g, the empty cylinder and a clear rigid striking plate which is larger than the surface of the cylinder. Fill the cylinder with tap water; apply the striking plate wiping all the exterior surfaces until dry and re-weigh. The difference between the two weighings should equal 1 000 g ± 30 g. Repeat the test, the difference between the two tests should not exceed ± 5 g.

A.3.2 **Removable collar**, 50 mm high and of the same internal diameter as the cylinder.

A.3.3 **Plunger**, having a diameter (d_1) 5 mm less than both the cylinder and the collar and having a mass as per Table A.1 to maintain a pressure of 9,17 g/cm^2.

A.3.4 **Funnel with a slope at approximately 60 °**, with a lower diameter to fit the collar.

A.3.5 **Supported fall controller:** Sieve with a perforated plate or woven wire screen with an approximate 200 mm diameter, 20 mm square apertures or 20 mm mesh size, independently supported approximately 5 mm above the funnel and not connected to it or the cylinder.

A.3.6 **Analytical balance**, with a scale interval of 1 g and a capacity of weighing 5 000 g.

A.3.7 **Straight edge.**

A.3.8 **Scoop,** 250 ml to 500 ml capacity.

A.3.9 **Tripod,** to support the fall controller (A.3.5).

A.4 Procedure

A.4.1 Manually, but carefully homogenize and gently open up any agglomerations or accretions that have resulted from handling or transportation of the sample. (8.1)

NOTE Care should be taken to avoid reducing the inherent particle size, which would render the material finer than it was at the time of manufacture.

A.4.2 Take about 5 l of the homogenized sample (A.4.1) and using the scoop (A.3.8), pass the material through the screen (A.3.5) gently agitating the material if required. If a more than 10 % volume is retained on the screen then the procedure is inappropriate for the material under test. If a less than 10 % volume is retained, break the material down as described in 8.3.

Asociación Española de Normalización, UNE
Este documento ha sido adquirido por Pedro Antonio Mejia Guerra el 2018-7-22.
Para poder utilizarlo en un sistema de red interno, deberá disponer de la correspodiente licencia de AENOR

A.4.3 Weigh the empty test cylinder (A.3.1) to the nearest 1 g using the balance (A.3.6). Place the collar (A.3.2) and the funnel (A.3.4) in position. Place the screen (A.3.5) in position approximately 5 mm above the funnel.

A.4.4 Gently spread out approximately 5 l of the screened homogenized material (A.4.2) once again and, using the scoop (A.3.8), take equal amounts of the material throughout the mass, filling the apparatus by sprinkling the material on top of the screen/flow breaker (A.3.5). Material from each aliquot not immediately passing through the screen may be gently agitated with finger tips to assist the process. Material of each aliquot retained on the screen (but not exceeding 10 % of the total volume) shall be reduced in size (by halving for a minimum number of times), by hand or with the aid of any simple tool so that it just passes the screen before the next aliquot is applied.

A.4.5 Once the apparatus has overfilled, remove the screen and strike off the excess material level with the top of the collar using the straightedge (A.3.7). Gently place the plunger (A.3.3) on the material, leave for 180 s ± 10 s, then carefully remove the plunger and collar taking care not to vibrate the cylinder, then use the straight edge (A.3.7) to strike off the material level with the top of the cylinder avoiding further compaction or disturbance. Weigh the material and cylinder to the nearest 1 g using the balance. Record the result (m_X).

If the material is very compressible and the top surface is found to be below the top of the cylinder when the plunger is removed after static compaction of 180 s ± 10 s, then compaction is inappropriate for the test material. In these circumstances repeat the procedure without applying the plunger to provide a measure of the un-compacted bulk density. Record if the plunger was not used and include that information in the test report (13).

Striking off may be difficult with very coarse, fibrous, woody or otherwise heterogeneous materials, and large pieces might have to be removed by hand, cut with a pair of scissors or broken off level with the top of the cylinder. Any resulting depressions on the surface of the cylinder should be filled from the remainder of the sample.

A.4.6 Repeat 3 times using fresh material each time to obtain a mean value.

A.5 Expression of results

Calculate the arithmetic mean of all the results obtained using the following equation:

$$m_L = \frac{\sum m_x}{n} \qquad \text{(A.1)}$$

where

m_L is the arithmetic mean mass in grams of sample and cylinder;

m_X is the mass in grams of the cylinder and sample;

Σm_X is the sum of the mass in grams of n replicates where n is the number of replicates.

The laboratory bulk density is given by the following equation:

$$L_D = \frac{m_L - m_0}{V} \qquad \text{(A.2)}$$

where

L_D is the laboratory bulk density in grams per litre;

Este documento ha sido adquirido por Pedro Antonio Mejia Guerra el 2018-7-22.
Para poder utilizarlo en un sistema de red interno, deberá disponer de la correspodiente licencia de AENOR

Asociación Española de Normalización, UNE

m_o the mass in grams of the empty test cylinder;

m_L is the arithmetic mean mass in grams of sample and cylinder;

V is the volume in litres of the test cylinder.

A.6 Use and storage of material

A.6.1 Because of the physical characteristics of material subjected to this procedure will have been altered, the material used in this test should not be used subsequently for the determination of any physical properties such as moisture content, particle size analysis or water retention. However, it may be used for other analyses where a dried sample is appropriate.

A.6.2 If not for immediate use the sample should be placed into a moisture-proof bag or container and the container sealed and stored below 5 °C but not frozen.

A.7 Precision - Compacted laboratory bulk density

The repeatability and reproducibility of the compacted laboratory bulk density content measurement in three separately prepared samples should be in accordance with Table B.2.

A summary of the results of an interlaboratory trial to determine the precision of the method in accordance with ISO 5725 [1] to [6] is given in Annex B.

NOTE The values derived from the interlaboratory trial may not be applicable to concentrations and matrices other than those given.

A.8 Test report

The test report shall contain the following information:

a) reference to this European Standard (EN 13040:2007);

b) all information necessary for complete identification of the sample;

c) results of the determination to the nearest 5 g per l;

d) details of any operations not specified in the European Standard or regarded as optional, as well as any factor which may have affected the results.

Asociación Española de Normalización, UNE

Este documento ha sido adquirido por Pedro Antonio Mejia Guerra el 2018-7-22.
Para poder utilizarlo en un sistema de red interno, deberá disponer de la correspodiente licencia de AENOR

Annex B
(informative)

Results of an interlaboratory trial to determine moisture content and laboratory compacted bulk density

An interlaboratory trial was organized in 1995 under the auspices of the European Committee for Standardization, to test the procedures specified in this European Standard.

In this trial the number of laboratories given in Table B.1 and Table B.2 determined the moisture content and the laboratory compacted bulk density in three types of samples.

Table B.1 — Summary of the results of an interlaboratory trial for the determination of moisture content

Sample	Unfertilized peat perlite	Coarse bark	Composted straw and domestic sewage
Number of laboratories retained after eliminating outliers	18	17	18
Number of outliers (laboratories)	1	2	1
Mean Value [% m/m]	61,34	60,03	53,31
Repeatability standard deviation, s_r [% m/m]	0,43	0,95	0,69
Repeatability relative standard deviation [%]	1,99	4,41	3,62
Repeatability limit, r = 2,8s_r [% m/m]	1,22	2,65	1,93
Reproducibility standard deviation ,s_R [% m/m]	1,34	1,60	1,52
Reproducibility relative standard deviation [%]	6,13	7,45	7,97
Reproducibility limit, R = 2,8s_R [% m/m]	3,76	4,47	4,25

Asociación Española de Normalización, UNE

Este documento ha sido adquirido por Pedro Antonio Mejia Guerra el 2018-7-22.
Para poder utilizarlo en un sistema de red interno, deberá disponer de la correspodiente licencia de AENOR

Table B.2 — Summary of the results of the interlaboratory trial for the determination of laboratory compacted bulk density

Sample	Unfertilized peat perlite	Composted coarse bark	Composted straw and domestic sewage
Number of laboratories retained after eliminating outliers	14	14	14
Number of outliers (laboratories)	0	0	0
Mean Value [g/l]	204,01	382,06	608,7
Repeatability standard deviation ,s_r [g/l]	1,82	4,31	4,30
Repeatability relative standard deviation (%)	2,50	3,16	1,98
Repeatability limit, r = 2,8s_r [g/l]	5,11	12,08	12,03
Reproducibility standard deviation ,s_R [g/l]	9,92	26,98	19,26
Reproducibility relative standard deviation (%)	13,62	19,77	8,86
Reproducibility limit, R = 2,8s_R [g/l]	27,78	75,53	53,94

Asociación Española de Normalización, UNE

Este documento ha sido adquirido por Pedro Antonio Mejia Guerra el 2018-7-22.
Para poder utilizarlo en un sistema de red interno, deberá disponer de la correspodiente licencia de AENOR

Bibliography

[1] ISO 5725:1994, Accuracy (trueness and precision) of measurement methods and results.

[2] ISO 5725-2:1994, Accuracy (trueness and precision) of measurement methods and results — Part 2: Basic method for the determination of repeatability and reproducibility of a standard measurement method.

[3] ISO 5725-3:1994, Accuracy (trueness and precision) of measurement methods and results — Part 3: Intermediate measures of the precision of a standard measurement method.

[4] ISO 5725-4:1994, Accuracy (trueness and precision) of measurement methods and results — Part 4: Basic methods for the determination of the trueness of a standard measurement method.

[5] ISO 5725-5:1998, Accuracy (trueness and precision) of measurement methods and results — Part 5: Alternative methods for the determination of the precision of a standard measurement method.

[6] ISO 5725-6:1994, Accuracy (trueness and precision) of measurement methods and results — Part 6: Use in practice of accuracy values.

[7] ISO 6206, Chemical products for industrial use — Sampling — Vocabulary.

Asociación Española de Normalización, UNE

Este documento ha sido adquirido por Pedro Antonio Mejia Guerra el 2018-7-22.
Para poder utilizarlo en un sistema de red interno, deberá disponer de la correspodiente licencia de AENOR

For information regarding the development of standards contact:

Asociación Española de Normalización
Génova, 6
28004 MADRID-España
Tel.: 915 294 900
info@une.org
www.une.org

For information regarding the sale and distribution:

AENOR INTERNACIONAL S.A.U.
Tel.: 914 326 160
normas@aenor.com
www.aenor.com

Spanish standardization body in:

Asociación Española de Normalización, UNE

Este documento ha sido adquirido por Pedro Antonio Mejia Guerra el 2018-7-22.
Para poder utilizarlo en un sistema de red interno, deberá disponer de la correspodiente licencia de AENOR
