# Supplementary material for: Evaluation of physicochemical properties and enzymatic activity of organic substrates during four crop cycles in soilless containers
Source: Food Sci Nutr. 2018 Oct 19;6(8):2066–78. doi: 10.1002/fsn3.757 (PMC6261231; doi:10.1002/fsn3.757)
Supplement: Supplementary file 2 [file FSN3-6-2066-s002.pdf]

Spanish standard

UNE-EN 13039

May 2012

Soil improvers and growing media

Determination of organic matter content and ash

This standard has been prepared by the Technical Committee CTN 142 *Fertilizers, liming materials and means of growing* the Secretariat of which is held by ANFFE.

Asociación Española
de Normalización
Génova, 6 - 28004 Madrid
915 294 900
info@une.org
www.une.org

Este documento ha sido adquirido por Pedro Antonio Mejia Guerra el 2018-7-22.
Para poder utilizarlo en un sistema de red interno, deberá disponer de la correspodiente licencia de AENOR

UNE-EN 13039

Soil improvers and growing media
Determination of organic matter content and ash

Mejoradores de suelo y sustratos de cultivo. Determinación del contenido en materia orgánica y de las cenizas.

Amendements du sol et supports de culture. Détermination de la matière organique et des cendres.

This standard is the official English version of EN 13039:2011.

This standard was published as UNE-EN 13039:2012, which is the definitive Spanish version.

The remarks to this document must be sent to:

Asociación Española de Normalización

Génova, 6
28004 MADRID-España
Tel.: 915 294 900
info@une.org
www.une.org
Depósito legal: M 13956:2018

© UNE 2018
Published by AENOR INTERNACIONAL S.A.U. under licence of Asociación Española de Normalización.
Forbidden reproduction

Asociación Española de Normalización, UNE

Este documento ha sido adquirido por Pedro Antonio Mejia Guerra el 2018-7-22.
Para poder utilizarlo en un sistema de red interno, deberá disponer de la correspodiente licencia de AENOR

EUROPEAN STANDARD
NORME EUROPÉENNE
EUROPÄISCHE NORM

EN 13039

November 2011

ICS 65.080

Supersedes EN 13039:1999

English Version

Soil improvers and growing media - Determination of organic matter content and ash

Amendements du sol et supports de culture - Détermination de la matière organique et des cendres

Bodenverbesserungsmittel und Kultursubstrate - Bestimmung des Gehaltes an organischer Substanz und Asche

This European Standard was approved by CEN on 17 September 2011.

CEN members are bound to comply with the CEN/CENELEC Internal Regulations which stipulate the conditions for giving this European Standard the status of a national standard without any alteration. Up-to-date lists and bibliographical references concerning such national standards may be obtained on application to the CEN-CENELEC Management Centre or to any CEN member.

This European Standard exists in three official versions (English, French, German). A version in any other language made by translation under the responsibility of a CEN member into its own language and notified to the CEN-CENELEC Management Centre has the same status as the official versions.

CEN members are the national standards bodies of Austria, Belgium, Bulgaria, Croatia, Cyprus, Czech Republic, Denmark, Estonia, Finland, France, Germany, Greece, Hungary, Iceland, Ireland, Italy, Latvia, Lithuania, Luxembourg, Malta, Netherlands, Norway, Poland, Portugal, Romania, Slovakia, Slovenia, Spain, Sweden, Switzerland and United Kingdom.

EUROPEAN COMMITTEE FOR STANDARDIZATION
COMITÉ EUROPÉEN DE NORMALISATION
EUROPÄISCHES KOMITEE FÜR NORMUNG

Management Centre: Avenue Marnix 17, B-1000 Brussels

© 2011 CEN All rights of exploitation in any form and by any means reserved worldwide for CEN national Members.

Ref. No. EN 13039:2011: E

Asociación Española de Normalización, UNE

Este documento ha sido adquirido por Pedro Antonio Mejia Guerra el 2018-7-22.
Para poder utilizarlo en un sistema de red interno, deberá disponer de la correspodiente licencia de AENOR

Contents

Page

Foreword....3
1 Scope....4
2 Normative references....4
3 Terms and definitions....4
4 Principle....4
5 Apparatus....4
6 Procedure....5
6.1 Test sample....5
6.2 Preparation of the basin....5
6.3 Determination....5
7 Calculation and expression of results....5
8 Precision....6
9 Test report....6
Annex A (informative) Results of an interlaboratory trial to determine the organic matter....7

Asociación Española de Normalización, UNE

Este documento ha sido adquirido por Pedro Antonio Mejia Guerra el 2018-7-22.
Para poder utilizarlo en un sistema de red interno, deberá disponer de la correspodiente licencia de AENOR

Foreword

This document (EN 13039:2011) has been prepared by Technical Committee CEN/TC 223 “Soil improvers and growing media”, the secretariat of which is held by ASI.

This European Standard shall be given the status of a national standard, either by publication of an identical text or by endorsement, at the latest by May 2012, and conflicting national standards shall be withdrawn at the latest by May 2012.

Attention is drawn to the possibility that some of the elements of this document may be the subject of patent rights. CEN [and/or CENELEC] shall not be held responsible for identifying any or all such patent rights.

This document supersedes EN 13039:1999.

The main change to the previous edition is in the scope.

According to the CEN/CENELEC Internal Regulations, the national standards organizations of the following countries are bound to implement this European Standard: Austria, Belgium, Bulgaria, Croatia, Cyprus, Czech Republic, Denmark, Estonia, Finland, France, Germany, Greece, Hungary, Iceland, Ireland, Italy, Latvia, Lithuania, Luxembourg, Malta, Netherlands, Norway, Poland, Portugal, Romania, Slovakia, Slovenia, Spain, Sweden, Switzerland and the United Kingdom.

Asociación Española de Normalización, UNE

Este documento ha sido adquirido por Pedro Antonio Mejia Guerra el 2018-7-22.
Para poder utilizarlo en un sistema de red interno, deberá disponer de la correspodiente licencia de AENOR

1 Scope

This European Standard specifies a routine method for determining the organic matter and the ash content of soil improvers and growing media.

This method is not applicable to liming materials and preformed materials such as mineral wool slabs and foam slabs.

The requirements of the standard may differ from the national legal requirements for the declaration of the products concerned.

2 Normative references

The following referenced documents are indispensable for the application of this document. For dated references, only the edition cited applies. For undated references, the latest edition of the referenced document (including any amendments) applies.

EN 12579:1999, *Soil improvers and growing media – Sampling*

EN 13040:2007, *Soil improvers and growing media – Sample preparation for chemical and physical tests, determination of dry matter content, moisture content and laboratory compacted bulk density*

ISO 5725 (all parts), *Accuracy (truenesss and precision) of measurement methods and results*

3 Terms and definitions

For the purposes of this document, the terms and definitions given in EN 12579:1999 and the following apply.

3.1
organic matter
carbon fraction of a sample which is free from water and inorganic substances

The organic matter for the purposes of this standard is taken as equal to loss on dry incineration at (450 ± 25) °C.

3.2
ash
residual mineral matter remaining after the destruction of organic matter/material by controlled burning

4 Principle

The test portion is dried at (103 ± 2) °C, then ashed at (450 ± 25) °C. The ash is determined as the residue on ignition. The organic matter is taken to be the loss of mass on ignition. Both are expressed as a percentage by mass of the dried sample.

5 Apparatus

5.1 **Drying oven**, capable of maintaining a temperature of (103 ± 2) °C.

5.2 **Electric muffle furnace**, capable of maintaining temperatures of (450 ± 25) °C and (550 ± 25) °C.

Asociación Española de Normalización, UNE

Este documento ha sido adquirido por Pedro Antonio Mejia Guerra el 2018-7-22.
Para poder utilizarlo en un sistema de red interno, deberá disponer de la correspodiente licencia de AENOR

5.3 Basin, made from fused silica or quartz, of shallow form with a flat bottom, capable of holding a sample of 5 g. Typical dimensions are 70 mm width and 20 mm height.

5.4 Desiccator containing an active drying agent.

5.5 Analytical balance with a scale interval 0,001 g.

6 Procedure

6.1 Test sample

Prepare the test sample in accordance with Clause 9 of EN 13040:2007.

6.2 Preparation of the basin

Heat the basin (see 5.3) for 16 h in the muffle furnace (see 5.2) at (550 ± 25) °C. Cool in the desiccator (see 5.4). After cooling, weigh to the nearest 0,001 g. Note the mass of the basin m_0.

NOTE The basin is heated to (550 ± 25) °C to ensure that all volatile material is removed prior to the test.

6.3 Determination

Spread evenly over the surface of the basin (see 5.3) approximately 5 g of the test sample (see 6.1) and dry in the oven (see 5.1) at a temperature of (103 ± 2) °C for 4 h.

Allow the basin and contents to cool to room temperature in the desiccator (see 5.4) and weigh to the nearest 0,001 g. Place the basin and contents in the oven (see 5.1) maintained at (103 ± 2) °C for a further 1 h.

Allow the basin and contents to cool to room temperature in the desiccator (see 5.4) and weigh to the nearest 0,001 g. Repeat the operations of heating, cooling and weighing until the difference between two successive weightings is less than 0,01 g. Note the mass of the basin and dried sample m_1.

Place the basin and contents in the cool muffle furnace (see 5.2) and raise the temperature over approximately 1 h to (450 ± 25) °C. Maintain this temperature for 6 h. Allow the basin and contents to cool to room temperature in the desiccator (see 5.4) and weigh to the nearest 0,001 g. Place the basin and contents into the muffle furnace (see 5.2) maintained at (450 ± 25) °C for a further 1 h.

Allow the basin and contents to cool to room temperature in the desiccator (see 5.4) and weigh to the nearest 0,001 g. Repeat the operations of heating, cooling and weighing until the difference between two successive weightings is less than 0,01 g. Note the mass of the basin and sample after ignition m_2.

7 Calculation and expression of results

The organic matter content, expressed as a percentage by mass of the dried sample, is given by the following equation:

$$W_{\mathrm{om}} = \frac{m_1 - m_2}{m_1 - m_0} \times 100 \qquad (1)$$

The ash content, expressed as a percentage by mass of the dried sample, is given by the following equation:

Asociación Española de Normalización, UNE

Este documento ha sido adquirido por Pedro Antonio Mejia Guerra el 2018-7-22.
Para poder utilizarlo en un sistema de red interno, deberá disponer de la correspodiente licencia de AENOR

$$W_{\text{ash}} = \frac{m_2 - m_0}{m_1 - m_0} \times 100 \qquad (2)$$

where

W_{om} is the organic matter content, in % m/m;

W_{ash} is the ash content, in % m/m;

m_0 is the mass of the basin, in g;

m_1 is the mass of the basin and the sample after drying in grams;

m_2 is the mass of the basin and the sample after ignition in grams.

8 Precision

The repeatability and reproducibility of the organic matter (*ash*) of the sample measured in 2 separately prepared samples should be in accordance with Table A.1.

A summary of the results of an interlaboratory trial to determine the precision of the method in accordance with ISO 5725 is given in Annex A.

The values derived from the interlaboratory trial may not be applicable to concentrations and matrices other than those given.

9 Test report

The test report shall include the following:

a) a complete identification of the sample;

b) a reference to this European Standard;

c) the results expressed in accordance with Clause 7;

d) any unusual features noticed during the determination;

e) details of any operation not specified in the European Standard or regarded as optional, as well as any factor which may have affected the results.

Asociación Española de Normalización, UNE

Este documento ha sido adquirido por Pedro Antonio Mejia Guerra el 2018-7-22.
Para poder utilizarlo en un sistema de red interno, deberá disponer de la correspodiente licencia de AENOR

Annex A
(informative)

Results of an interlaboratory trial to determine the organic matter

An interlaboratory trial was organized in 1995 under the auspices of the European Committee for Standardization, to test the procedures specified in this European Standard.

In this trial the number of laboratories given in Table A.1 determined the organic matter in three types of samples.

Table A.1 — Summary of the results of the interlaboratory trial for the determination of organic matter

Sample	Unfertilized peat perlite	Composted coarse bark	Sewage sludge composted with straw
Number of laboratories retained after eliminating outliers	19	18	19
Number of outliers (laboratories)	0	1	0
Mean Value (m/m %)	79,86	76,03	44,34
Repeatability standard deviation, s_r (% m/m)	1,00	1,29	1,20
Repeatability relative standard deviation (% m/m)	1,25	1,70	2,71
Repeatability limit, r = 2,8 s_r (% m/m)	2,8	3,60	3,37
Reproducibility standard deviation, s_R (% m/m)	1,51	2,33	2,26
Reproducibility relative standard deviation (%)	1,89	3,06	5,10
Reproducibility limit, R = 2,8 s_R (% m/m)	4,23	6,51	6,33

Asociación Española de Normalización, UNE

Este documento ha sido adquirido por Pedro Antonio Mejia Guerra el 2018-7-22.
Para poder utilizarlo en un sistema de red interno, deberá disponer de la correspodiente licencia de AENOR

For information regarding the development of standards contact:

Asociación Española de Normalización
Génova, 6
28004 MADRID-España
Tel.: 915 294 900
info@une.org
www.une.org

For information regarding the sale and distribution:

AENOR INTERNACIONAL S.A.U.
Tel.: 914 326 160
normas@aenor.com
www.aenor.com

Spanish standardization body in:

Asociación Española de Normalización, UNE

Este documento ha sido adquirido por Pedro Antonio Mejia Guerra el 2018-7-22.
Para poder utilizarlo en un sistema de red interno, deberá disponer de la correspodiente licencia de AENOR
